# HSD3B1 is an oxysterol 3β-hydroxysteroid dehydrogenase in human placenta

**DOI:** 10.1098/rsob.220313

**Published:** 2023-05-03

**Authors:** Alison Dickson, Eylan Yutuc, Catherine A. Thornton, James E. Dunford, Udo Oppermann, Yuqin Wang, William J. Griffiths

**Affiliations:** ^1^ Swansea University Medical School, ILS1 Building, Singleton Park, Swansea SA2 8PP, UK; ^2^ Centre for Translational Myeloma Research, NIHR Biomedical Research Centre, Botnar Research Centre, University of Oxford, Oxford OX3 7LD, UK

**Keywords:** HSD3B1, oxysterol, placenta, mass spectrometry, bile acid, pregnancy

## Abstract

Most biologically active oxysterols have a 3β-hydroxy-5-ene function in the ring system with an additional site of oxidation at C-7 or on the side-chain. In blood plasma oxysterols with a 7α-hydroxy group are also observed with the alternative 3-oxo-4-ene function in the ring system formed by ubiquitously expressed 3β-hydroxy-Δ^5^-C_27_-steroid oxidoreductase Δ^5^-isomerase, HSD3B7. However, oxysterols without a 7α-hydroxy group are not substrates for HSD3B7 and are not usually observed with the 3-oxo-4-ene function. Here we report the unexpected identification of oxysterols in plasma derived from umbilical cord blood and blood from pregnant women taken before delivery at 37+ weeks of gestation, of side-chain oxysterols with a 3-oxo-4-ene function but no 7α-hydroxy group. These 3-oxo-4-ene oxysterols were also identified in placenta, leading to the hypothesis that they may be formed by a previously unrecognized 3β-hydroxy-Δ^5^-C_27_-steroid oxidoreductase Δ^5^-isomerase activity of HSD3B1, an enzyme which is highly expressed in placenta. Proof-of-principle experiments confirmed that HSD3B1 has this activity. We speculate that HSD3B1 in placenta is the source of the unexpected 3-oxo-4-ene oxysterols in cord and pregnant women's plasma and may have a role in controlling the abundance of biologically active oxysterols delivered to the fetus.

## Graphical abstract

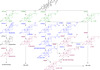

## 1. Introduction

**Figure 1.**
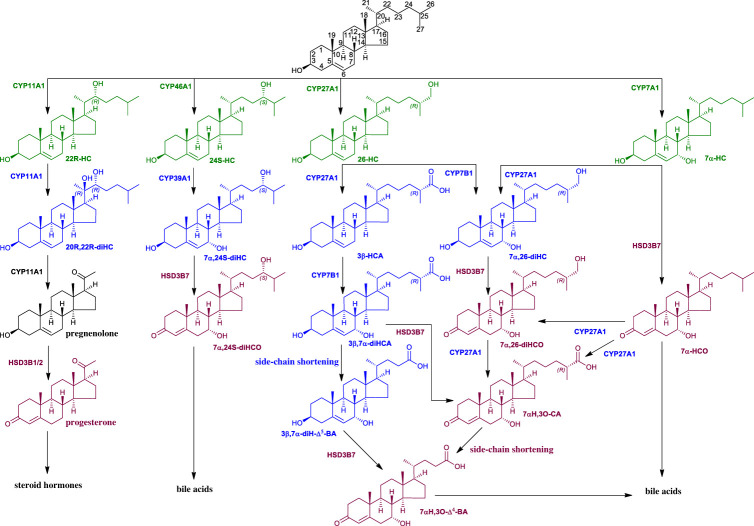
Abbreviated scheme of oxysterol metabolism. Primary oxysterols (hydroxycholesterols, HC) are in green, secondary oxysterols (diHC), including 3β-hydroxycholestenoic acids, are in blue and 3-oxo-4-ens are in claret.

Oxysterols are oxidized forms of cholesterol or of its precursors [1]. The primary routes of oxysterol metabolism are 7α-hydroxylation catalysed by cytochrome P450 (CYP) 7B1 [2], or in the specific case of 24S-hydroxycholesterol (24S-HC) by CYP39A1 [3], and (25R)26-hydroxylation or carboxylation catalysed by CYP27A1 (figure 1) [4–6]. Once 7α-hydroxylated, oxysterols become substrates for the ubiquitous hydroxysteroid dehydrogenase (HSD) 3B7 [7–9], which oxidizes the 3β-hydroxy group to a 3-ketone and isomerizes the double bond from Δ^5^ to Δ^4^ (figure 1), a key reaction in bile acid biosynthesis necessary for conversion of initial 3β-hydroxy stereochemistry, as in the cholesterol structure, to the 3α-hydroxy stereochemistry in primary bile acids [5,6,10,11]. Cholesterol itself is 7α-hydroxylated by CYP7A1 to 7α-hydroxycholesterol (7α-HC) [12], which like other oxysterols with a 7α-hydroxy group, is a substrate for HSD3B7 [5]. With respect to oxysterols, including down-stream sterol-acids, oxidation at C-3 with accompanying Δ^5^–Δ^4^ isomerization can be regarded as a deactivation mechanism eliminating many of the biological activities of the substrate oxysterol. This is the case for the chemoattractant oxysterols 7α,25-dihydroxycholesterol (7α,25-diHC) and 7α,(25R)26-dihydroxycholesterol (7α,26-diHC, also known as 7α,27-dihydroxycholesterol) [13], and the liver X receptor (LXRα and LXRβ) ligand 3β,7α-dihydroxycholest-5-en-(25R)26-oic acid (3β,7α-diHCA) [14]. Note that in much of the literature (25R)26-hydroxylation and (25R)26-carboxylation are described according to non-systematic nomenclature as 27-hydroxylation and 27-carboxylation, with the stereochemistry assumed to be 25R [15]. Here we prefer to use systematic numbering, and for brevity when hydroxylation or carboxylation is at C-26 the reader can assume that the stereochemistry is 25R unless stated otherwise, i.e. 26-HC is used as the abbreviation for (25R)26-hydroxycholesterol. While HSD3B7 has activity towards 7α-hydroxyoxysterols it has not been reported to oxidize oxysterols lacking the 7α-hydroxy group [8]. Nevertheless deficiency in HSD3B7 does not completely eliminate primary bile acid production [16], suggesting that a second HSD may have 3β-hydroxy-Δ^5^-C_27_-steroid oxidoreductase activity in human.

In humans, the HSD3B1 and HSD3B2 enzymes belong to the evolutionarily conserved superfamily of short-chain dehydrogenases/reductases (SDR) [17], with official nomenclature symbols SDR11E1 and SDR11E2, respectively [18]. They convert C_19_ and C_21_ steroids with a 3β-hydroxy-5-ene structure to 3-oxo-4-ene products (figure 1). HSD3B1 is primarily localized to placenta and non-steroidogenic tissue, while HSD3B2 is primarily expressed in the adrenal gland, ovary and testis [9,19]. The 3β-hydroxy-5-ene to 3-oxo-4-ene transformation is an essential step in the biosynthesis of all classes of active steroid hormones [20,21]. HSD3B1/2 enzymes are not reported to use C_27_ steroids as substrates although they share about 34% sequence identity to HSD3B7 (SDR11E3) in human, and HSD3B7 does not use C_19_ or C_21_ steroids as substrates suggesting different physiological roles for these HSD enzymes [8].

Two side-chain oxysterols that have not been reported to be 7α-hydroxylated *in vivo* are 22R-hydroxycholesterol (22R-HC) and 20S-hydroxycholesterol (20S-HC). Instead, 22R-HC becomes hydroxylated to 20R,22R-dihydroxycholesterol (20R,22R-diHC) which then undergoes side-chain shortening to pregnenolone in reactions catalysed by CYP11A1 (figure 1) [22,23]. 20S-HC has also been reported to be converted to pregnenolone [1]. Pregnenolone is converted by HSD3B1/2 enzymes to progesterone. 20S-HC, 22R-HC and 20R,22R-diHC are ligands to LXRs [24–26], while 22R-HC, like many other side-chain hydroxylated 3β-hydroxysterols, is a ligand to INSIG (insulin induced gene), important in the regulation of SREBP-2 (sterol regulatory element-binding protein-2) processing and cholesterol biosynthesis [27]. 20S-HC also regulates SREBP-2 processing [28], presumably by binding to INSIG, and is a ligand to the G protein-coupled receptor (GPCR) Smoothened (SMO) important in the Hedgehog (Hh) signalling pathway [29], and has recently been reported to be a ligand to the sigma 2 (*σ*2) receptor TMEM97 [30]. These biological activities are not known to be conveyed to the 3-oxo-4-ene analogues of 20S-HC, 22R-HC or 20S,22R-diHC, again suggesting that oxidation at C-3 with accompanying Δ^5^–Δ^4^ isomerization may be a deactivation mechanism of oxysterols.

While oxysterols, including sterol-acids, based on a 3β-hydroxy-5-ene framework are routinely analysed by both gas chromatography–mass spectrometry (GC-MS) [31–33] and liquid chromatography (LC)–MS [31,32,34–36], with the exception of 7α-hydroxycholest-4-en-3-one (7α-HCO) and 7α-hydroxy-3-oxocholest-4-en-(25R/S)26-oic acid (7αH,3O-CA), this is not normally the case for the 3-oxo-4-ene sterols [31,37–39]. The ‘enzyme-assisted derivatization for sterol analysis' (EADSA) technology, as used in the current study, allows the analysis of 3β-hydroxy-5-ene and 3-oxo-4-ene oxysterols, including sterol-acids, in a single LC-MS run [31,40–43]. In this technology, after extraction each sample is split into two equal aliquots: to the B-fraction endogenous 3-oxo-4-ene sterols are reacted with the [^2^H_0_]Girard P hydrazine (GP) reagent to tag a charge to the sterol skeleton to enhance sensitivity of LC-MS analysis; while to the A-fraction bacterial cholesterol oxidase enzyme is added. This converts sterols with a 3β-hydroxy-5-ene function to their 3-oxo-4-ene equivalents which are then reacted with [^2^H_5_]GP reagent (see electronic supplementary material, figure S1). A salient feature of the method is that *in fraction-B only sterols with a natural oxo group are derivatized* (*with [^2^H_0_]GP*), while *in fraction-A sterols with a natural oxo group and those oxidized by cholesterol oxidase to contain one are derivatized* (*with [^2^H_5_]GP*). The derivatization products are then combined and analysed by LC-MS, the originating structure of sterol, whether 3-oxo-4-ene or 3β-hydroxy-5-ene, is revealed by the isotope labelling of the GP reagents and deconvolution of the resultant data from A- and B-fractions [40].

3β-Hydroxycholest-5-en-(25R)26-oic acid (3β-HCA) does not have a 7α-hydroxy group and is a C_27_ sterol so should not be a substrate for HSD3B7 or HSD3B1/2 enzymes; however, 3-oxocholest-4-en-(25R)26-oic acid (3O-CA) is found at low levels in human plasma at about 1–5% of 3β-HCA [31,41–43]. Here we report evidence for the presence of (25R)26-hydroxycholest-4-en-3-one (26-HCO), a potential precursor of 3O-CA, in human plasma and its elevated concentration in plasma from pregnant women taken 1–2 days prior to elective caesarean section at 37+ weeks of gestation and in plasma generated from blood of the umbilical cord. Besides 26-HCO and 3O-CA, we identify other C_27_–C_24_ cholesterol metabolites with a 3-oxo-4-ene structure but without 7α-hydroxy group in these plasmas and in human placenta. HSD3B1 is abundant in human placenta [19], and we present proof-of-principle data that demonstrate that the HSD3B1 enzyme will convert monohydroxycholesterols, where the added hydroxy group is in the side-chain, into hydroxycholest-4-en-3-ones.

## 2. Material and methods

### 2.1. Materials

The source of all materials for oxysterol analysis can be found in [43].

### 2.2. Human material

Maternal blood was taken 24–48 h prior to elective caesarean section at 37+ weeks of gestation for reasons that did not include maternal or fetal anomaly. Umbilical cord blood and placenta were collected at delivery of the baby. Control plasma was from non-pregnant females. All samples were collected with approval from a Health Research Authority Research Ethics Committee (approval numbers 11/WA/0040 and 13/WA/019). All participants provided informed consent and the study adhered to the principles of the Declaration of Helsinki.

### 2.3. Oxysterol analysis

Oxysterol analysis was performed as described in detail in [41–44] with minor modifications. In brief, oxysterols were extracted in acetonitrile (absolute ethanol in the case of placental tissue) and following dilution isolated by solid phase extraction (SPE). The extract was split into two equal fractions: A and B. Cholesterol oxidase was added to fraction-A to convert endogenous 3β-hydroxy-5-ene oxysterols to their 3-oxo-4-ene equivalents which were then derivatized with [^2^H_5_]GP (electronic supplementary material, figure S1). Fraction-B was treated with [^2^H_0_]GP in the *absence of cholesterol oxidase*, thus giving a measure of endogenous 3-oxo-4-ene oxysterols, while fraction-A gives a measure of endogenous 3β-hydroxy-5-ene plus 3-oxo-4-ene oxysterols. Fractions-A and -B were combined and analysed by LC-MS with multi-stage fragmentation (MS^3^), i.e. [M]^+^ → [M-Py]^+^→, on an Orbitrap Elite high resolution mass spectrometer (RRID:SCR_020548, ThermoFisher Scientific). In the MS mode resolution was 120 000 FWHM (full width at half maximum height) at *m*/*z* 400 with mass accuracy better than 5 ppm. MS^3^ spectra were recorded in the linear ion trap (LIT) in parallel to acquisition of mass spectra in the Orbitrap. Oxysterols were identified by reference to authentic standards, unless stated otherwise. Quantification was achieved with the isotope-labelled standards [25,26,26,26,27,27,27-^2^H_7_]24R/S-HC and [25,26,26,26,27,27,27-^2^H_7_]22R-hydroxycholest-4-en-3-one ([^2^H_7_]22R-HCO) which have been shown to be suitable for quantification of side-chain oxysterols [40,43]. Further details of the experimental methods can be found in electronic supplementary material.

### 2.4. Transfection studies

HSD3B1 transfection was performed using the pcDNA3-HSD3B1-STOP plasmid (see electronic supplementary material). The plasmid construct was transfected into HEK293 cells using JetOPTIMUS transfection reagent. Plasmid DNA was combined with JetOPTIMUS buffer at 1 µl per 10 ng of DNA and vortexed briefly. The JetOPTIMUS transfection reagent was added at 1 µl per 1000 ng of plasmid DNA and mixed gently. The mixture was incubated at room temperature for 10 min before pipetting evenly into seeded 60 mm dishes. The cells were incubated for 24 h at 37°C and 5% CO_2_ to allow for transfection and *in vitro* expression of the HSD3B1 enzyme from the HSD3B1 open reading frame (ORF) in the plasmid.

Transfected HEK293 cells were treated with oxysterols. The incubation buffer was potassium phosphate, pH 6.8, containing 1 mM EDTA, 1 mM of NAD^+^ and 1 µM of oxysterol. One millilitre of oxysterol incubation buffer was added to each dish. The cells were incubated for 1 h at 37°C, 5% CO_2_. An aliquot of cells was taken for immunoblot (100 µl of protein lysate loaded on gel; see electronic supplementary material) while a separate aliquot was taken for LC-MS(MS^3^) analysis.

## 3. Results

### 3.1. Plasma from pregnant women, plasma from umbilical cord blood and placental tissue contain 3-oxocholest-4-en-(25R)26-oic acid and (25R)26-hydroxycholest-4-en-3-one

**Figure 2.**
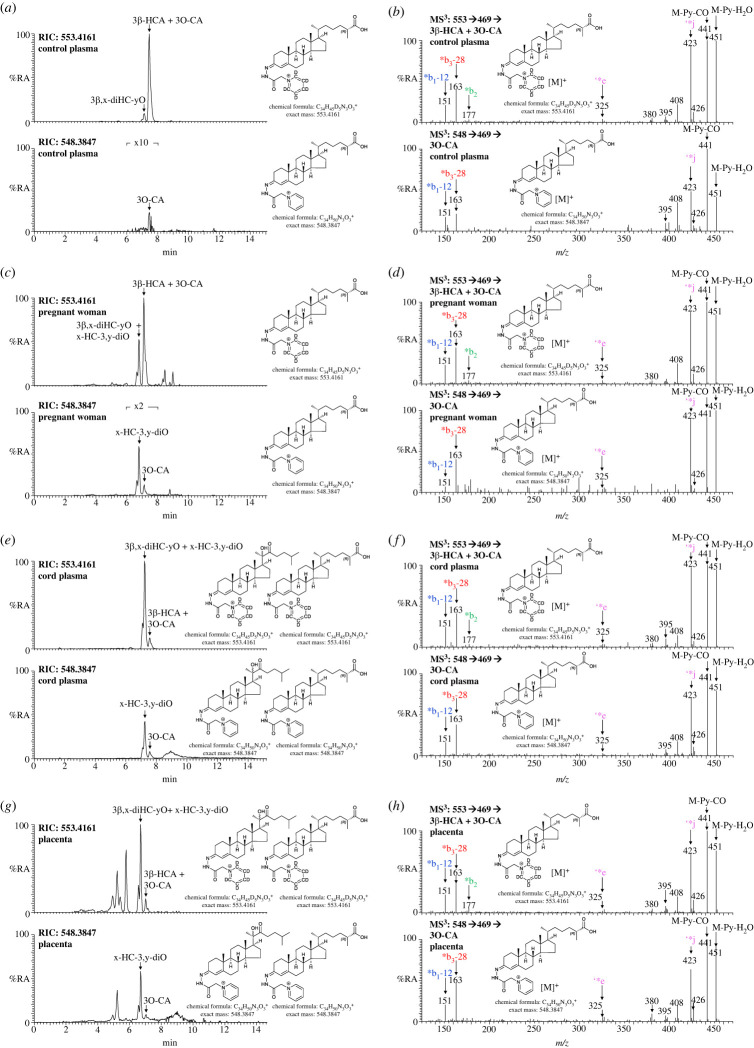
3β-HCA and 3O-CA are present in plasma from non-pregnant (control) and pregnant women, umbilical cord plasma and in placental tissue. Reconstructed ion chromatograms (RICs) of 553.4161 ± 5 ppm corresponding to 3β-HCA plus 3O-CA derivatized with [^2^H_5_]GP (upper panels) and 548.3847 ± 5 ppm corresponding to 3O-CA derivatized with [^2^H_0_]GP (lower panels). (*a*) Non-pregnant woman's (control) plasma, (*c*) pregnant woman's plasma, (*e*) cord plasma, and (*g*) placenta. Chromatograms in upper and lower panels are plotted on the same *y*-axis and magnified as indicated. MS^3^ ([M]^+^ → [M-Py^+^→) spectra of 3β-HCA plus 3O-CA derivatized with [^2^H_5_]GP (upper panels) and 3O-CA derivatized with [^2^H_0_]GP (lower panels) from (*b*) non-pregnant woman's (control) plasma, (*d*) pregnant woman's plasma, (*f*) cord plasma, and (*h*) placenta. There is some shift in retention time between samples which were analysed at different times on different LC columns but of the same type. MS^3^ spectra can be compared to those of authentic standards [43]. Further data can be found in electronic supplementary material, figure S2A–D.

The sterol-acid 3O-CA has been identified previously at low levels compared with 3β-HCA in human plasma. Its origin is unknown. As part of an investigation into oxysterols, including sterol-acids, associated with human pregnancy EADSA technology was employed to identify oxysterols based on their 3β-hydroxy-5-ene scaffold or native oxo group. As anticipated, 3O-CA was found in blood from pregnant women taken 1–2 days before elective caesarean section, but at quite appreciable levels (2.41 ± 0.61 ng ml^−1^ ± s.d.), corresponding to about 6% of that of 3β-HCA (figure 2*c*,*d* and table 1; see also electronic supplementary material, figure S2B). In terms of absolute concentration 3O-CA was found at similar levels in plasma from non-pregnant women (control plasma, 1.99 ± 0.69 ng ml^−1^), but when compared with 3β-HCA at a level of only 2% (figure 2*a*,*b*; electronic supplementary material, figure S2A). Cord blood is blood left over in the placenta after birth and collected from the umbilical cord, the level of 3O-CA in cord plasma (12.74 ± 6.60 ng ml^−1^) is higher than in circulating plasma from both pregnant and non-pregnant women, and similar to that of 3β-HCA in cord plasma (figure 2*e*,*f*; electronic supplementary material, figure S2C). These data suggest that 3O-CA may be produced from 3β-HCA in the placenta, where the levels of 3O-CA (3.99 ± 1.29 ng g^−1^) are about the same as those of 3β-HCA (figure 2*g*,*h*; electronic supplementary material, figure S2D). Note that concentrations of 3β-HCA are reported in [44].

**Table 1.** 3β-Hydroxy-5-ene and 3-oxo-4-ene sterols in plasma from non-pregnant and pregnant women and the umbilical cord. Data for 22R-HC, 24S-HC, 26-HC, 7α,26-diHC and 3β-HCA are reported in [44]. Control plasma is plasma from non-pregnant females. Note: 1. Single outlier removed from control 25-HC data. 2. Presumptive identification in the absence of authentic standard. Data only semi-quantitative. 3. Generic structure 3β,x-diHC-yO. 4. Generic structure x-HC,3,y-diO. 5. Generic structure 3β,y-diHCA. 6. Generic structure yH,3O-CA. 7. Generic structure 3β,x-diHCA. 8. Generic structure xH,3O-CA.

[^2^H_0_]GP	[^2^H_5_]GP	compound	cord plasma (*n* = 14)			pregnant woman plasma (*n* = 10)			control plasma (*n* = 5)			note
*m/z*	*m/z*	abbreviation	mean (ng ml^−1^)	s.d.	3-one/3β-OL	mean (ng ml^−1^)	s.d.	3-one/3β-OL	mean (ng ml^−1^)	s.d.	3-one/3β-OL	
	539.4368	22R-HC	6.19	3.01		2.55	1.18		0.00	0.00		
534.4054		22R-HCO	0.11	0.21	0.02	0.00	0.00	0.00	0.00	0.00	0.00	
	539.4368	24S-HC	7.34	2.38		14.99	4.28		14.48	3.33		
534.4054		24S-HCO	0.29	0.39	0.04	0.04	0.10	0.00	0.00	0.00	0.00	
	539.4368	25-HC	2.75	1.88		2.74	1.66		1.55	0.13		1
534.4054		25-HCO	0.00	0.00	0.00	0.00	0.00	0.00	0.00	0.00	0.00	
	539.4368	26-HC	7.23	2.88		21.61	5.63		25.67	2.30		
534.4054		26-HCO	1.65	0.68	0.23	0.69	0.42	0.03	0.00	0.00	0.00	
												
	555.43172	7α,25-diHC	0.48	0.48		1.07	0.35		0.79	0.64		
550.40032		7α,25-diHCO	0.72	0.88	1.50	1.77	0.76	1.65	1.40	0.43	1.77	
	555.43172	7α,26-diHC	0.49	0.23		1.03	0.54		1.08	0.55		
550.40032		7α,26-diHCO	3.24	1.26	6.56	3.89	1.19	3.79	4.13	1.23	3.82	
	555.43172	20R,22R-diHC	49.74	23.97		13.23	6.79		0.00	0.00		
550.40032		20R,22R-diHCO	12.86	6.09	0.26	6.04	3.94	0.46	0.00	0.00	0.00	
												
	553.41607	3β-HCA	10.56	4.71		43.64	13.71		90.02	21.40		
548.38467		3O-CA	12.74	6.60	1.21	2.41	0.61	0.06	1.99	0.69	0.02	
	511.3691	3βH-Δ^5^-BA	3.37	2.64		1.50	0.81		1.89	0.65		
506.3377		3O-Δ^4^-BA	0.76	0.47	0.23	0.00	0.00	0.00	0.00	0.00	0.00	
												
	553.41607	3β,20-diHC-22O	103.60	44.51		21.72	8.22		10.59	5.77		2, 3
548.38467		20-HC-3,22-diO	78.24	30.85	0.76	18.21	6.91	0.84	0.00	0.00	0.00	2, 4
	569.41098	3β,25-diHCA	13.02	6.47		6.34	2.03		6.36	3.05		2, 5
564.37958		25H,3O-CA	12.12	5.03	0.93	4.29	2.64	0.68	0.00	0.00	0.00	2, 6
	569.41098	3β,x-diHCA	34.00	16.87		7.42	2.95		4.68	1.82		2, 7
564.37958		xH,3O-CA	11.80	4.60	0.35	4.99	2.25	0.67	0.00	0.00		2, 8

**Figure 3.**
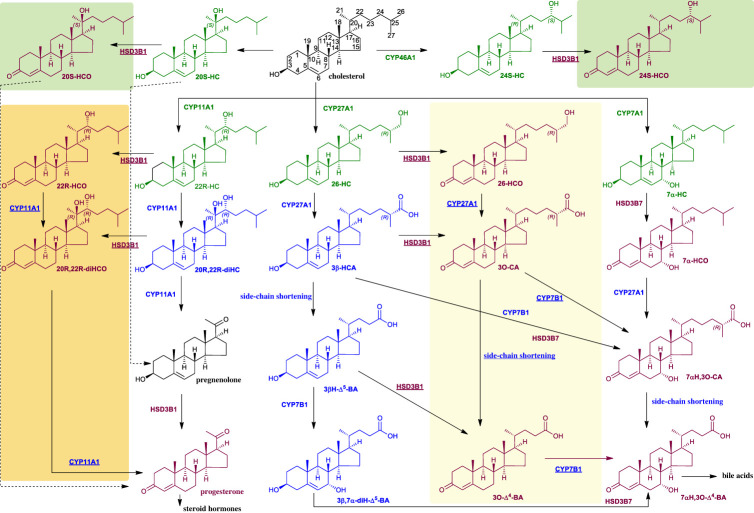
Suggested metabolic pathways generating oxysterols with a 3-oxo-4-ene function. Unexpected pathways are shown on pale-yellow, orange and lime backgrounds. Oxysterols and steroids are coloured as in figure 1.

**Figure 4.**
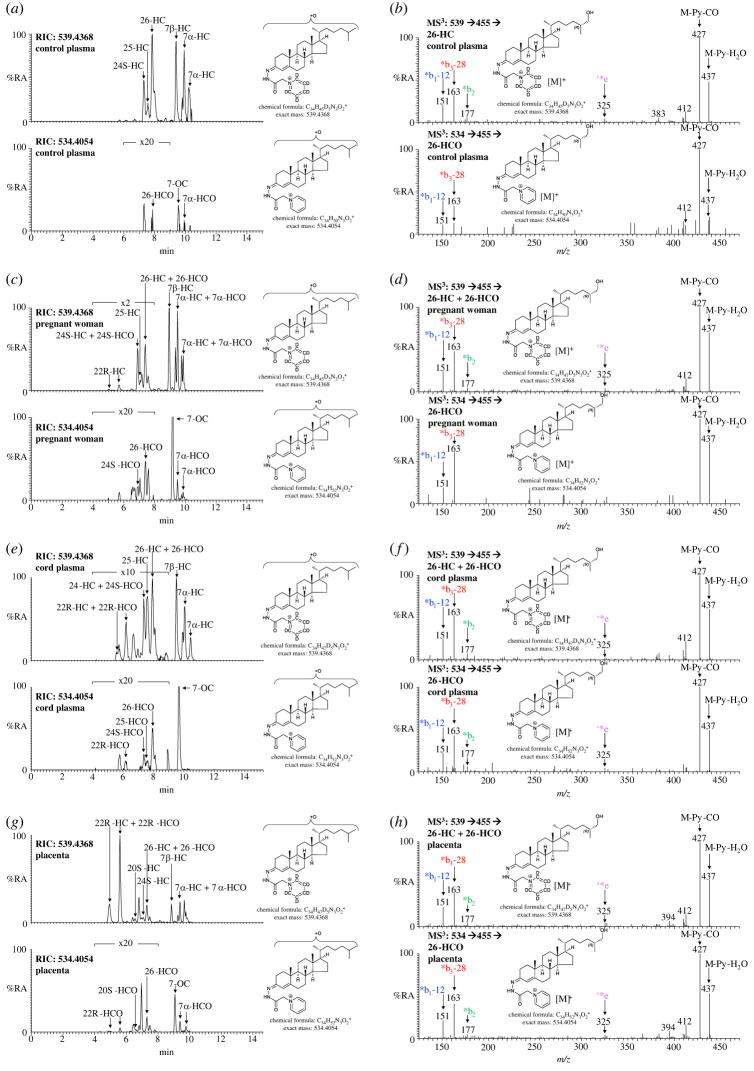
Monohydroxycholesterols (HC) and monohydroxycholestenones (HCO) are present in plasma from non-pregnant (control) and pregnant women, umbilical cord plasma and in placental tissue. RICs of 539.4368 ± 5 ppm corresponding to monohydroxycholesterols plus monohydroxycholestenones derivatized with [^2^H_5_]GP (upper panels) and 534.4054 ± 5 ppm corresponding to monohydroxycholestenones derivatized with [^2^H_0_]GP (lower panels). (*a*) Non-pregnant woman's (control) plasma, (*c*) pregnant woman's plasma, (*e*) cord plasma, and (*g*) placenta. Chromatograms in upper and lower panels are plotted on the same *y*-axis and magnified as indicated. MS^3^ ([M]^+^ → [M-Py]^+^→) spectra of 26-HC plus 26-HCO derivatized with [^2^H_5_]GP (upper panels) and 26-HCO derivatized with [^2^H_0_]GP (lower panels) from (*b*) non-pregnant woman's (control) plasma, (*d*) pregnant woman's plasma, (*f*) cord plasma, and (*h*) placenta. There is some shift in retention time between samples which were analysed at different times on different LC columns but of the same type. MS^3^ spectra can be compared to those of authentic standards [43]. Further data can be found in electronic supplementary material, figure S2E–H.

3O-CA may be derived by oxidation of 3β-HCA at C-3 or conceivably by oxidation of 26-HCO at C-26 to yield the carboxylic acid group (figure 3). Although to the best of our knowledge concentrations of 26-HCO have not been reported in plasma, we have seen evidence in previous studies for the presence of 26-HCO at about the level of detection but below the level of quantification (less than 0.2 ng ml^−1^). By investigating the reconstructed ion chromatogram (RIC) at *m*/*z* 534.4054 ± 5 ppm appropriate to 26-HCO following [^2^H_0_]GP-derivatization, a peak with the correct retention time is evident in plasma from pregnant women and co-eluting with that of 26-HC following *ex vivo* cholesterol oxidase treatment and [^2^H_5_]GP derivatization at *m*/*z* 539.4368 ± 5 ppm (figure 4*c*; see also electronic supplementary material, figure S2F). The MS^3^ ([M]^+^ → [M-Py]^+^→) fragmentation spectra of GP-derivatized 26-HCO and 26-HC are very similar, with the exception of the precursor-ion *m*/*z*, confirming the identification of endogenous 26-HCO (figure 4*d*; see Yutuc *et al.* [43] for MS^3^ spectra of reference standards). The level of 26-HCO in pregnant women's plasma is low at 0.69 ± 0.42 ng ml^−1^ and was only detected in nine of the ten samples analysed, being present at 3% that of 26-HC. In plasma from non-pregnant females, 26-HCO was only just detectable (figure 4*a*,*b*) but was below the limit of quantification (0.2 ng ml^−1^) in all samples analysed. The situation in cord plasma is quite different (figure 4*e*,*f*): the level of 26-HCO was found to be 1.65 ± 0.68 ng ml^−1^, 23% that of 26-HC. As was the case in placenta for 3O-CA and 3β-HCA, the ratio of 26-HCO to 26-HC in this tissue was also high, with 26-HCO at 16.0 ± 3.39 ng g^−1^ being 39% that of 26-HC (figure 4*g*,*h*).

### 3.2. Potential artefactual formation of 3-oxo-4-ene oxysterols from 3β-hydroxy-5-ene oxysterols

An alternative explanation for the current data showing the existence of 3-oxo-4-ene oxysterols formed in the absence of a 7α-hydroxy group is that their defining LC-MS peaks may be artefacts generated as a consequence of the presence of contaminating [^2^H_0_]GP within the [^2^H_5_]GP reagent which is used in conjunction with cholesterol oxidase enzyme in the EADSA process. Such a situation would result in a fraction of 3β-hydroxy-5-ene sterols becoming derivatized with [^2^H_0_]GP instead of exclusively by [^2^H_5_]GP and being incorrectly interpreted as being derived from endogenous 3-oxo-4-ene oxysterols. This situation is unlikely to be significant as the [^2^H_5_]pyridine from which [^2^H_5_]GP reagent is prepared is 99.94% isotopically pure, which should lead to no more than 0.06% artefactual formation of the [^2^H_0_]GP derivatives of 26-HCO or 3O-CA derived from native 26-HC and 3β-HCA, respectively. The levels of 26-HCO in pregnant women's and cord plasma are 3% and 23% of 26-HC, respectively, and in placenta 26-HCO is 39% of 26-HC, values very much greater than 0.06%. Another possibility whereby unreliable data can be generated is through back-exchange of the derivatization groups when fraction-A ([^2^H_5_]GP) and fraction-B ([^2^H_0_]GP) are mixed just prior to LC-MS analysis. However, in the absence of an acid catalyst the exchange reaction does not proceed. To confirm that the observations of unexpected 3-oxo-4-ene oxysterols were not *ex vivo* artefacts of sample preparation, including the EADSA process or of sample injection, the formation of [^2^H_7_]24R/S-hydroxycholest-4-en-3-one ([^2^H_7_]24R/S-HCO) from [^2^H_7_]24R/S-hydroxycholesterol ([^2^H_7_]24R/S-HC) internal standard, added during the first step of oxysterol extraction, was monitored for every sample analysed. In no case was the [^2^H_0_]GP derivative of [^2^H_7_]24R/S-HCO observed (electronic supplementary material, figure S3), eliminating the possibility of *ex vivo* formation of 3-oxo-4-ene oxysterols during sample preparation and analysis and confirming the high isotopic purity of [^2^H_5_]GP-hydrazine.

### 3.3. Metabolic products of 3-oxocholest-4-en-(25R)26-oic acid

**Figure 5.**
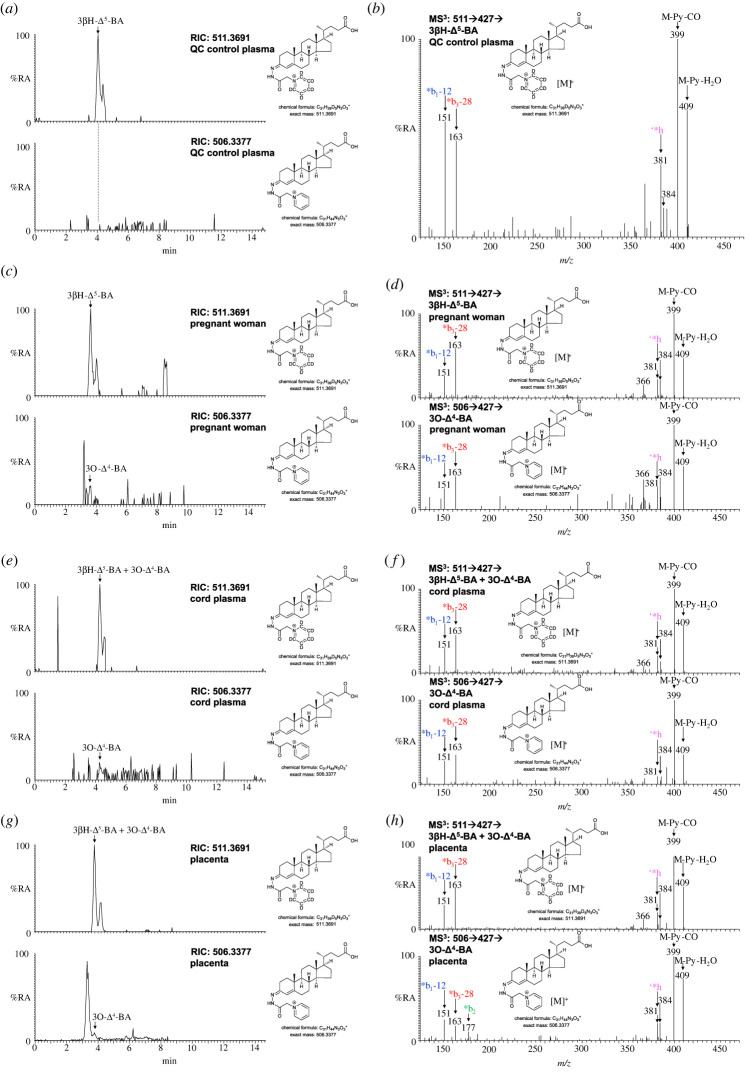
3-Oxo-4-ene C_24_-bile acids in pregnant women's and cord plasma and in placental tissue. RICs of 511.3691 ± 5 ppm corresponding to 3βH-Δ^5^-BA plus 3O-Δ^4^-BA (where present) derivatized with [^2^H_5_]GP (upper panels) and 506.3377 ± 5 ppm corresponding to 3O-Δ^4^-BA derivatized with [^2^H_0_]GP (lower panels). (*a*) Non-pregnant woman's (control) plasma, (*c*) pregnant woman's plasma, (*e*) cord plasma, and (*g*) placenta. Chromatograms in upper and lower panels are plotted on the same *y*-axis. MS^3^ ([M]^+^ → [M-Py]^+^→) spectra of 3βH-Δ^5^-BA plus 3O-Δ^4^-BA (if present) derivatized with [^2^H_5_]GP (upper panels) and where present 3O-Δ^4^-BA derivatized with [^2^H_0_]GP (lower panels) from (*b*) non-pregnant woman's (control) plasma, (*d*) pregnant woman's plasma, (*f*) cord plasma and (*h*) placenta. There is some shift in retention time between samples which were analysed at different times and on different LC columns but of the same type. MS^3^ spectra can be compared to those of authentic standards [43].

The immediate metabolic product of 3β-HCA in bile acid biosynthesis is 3β,7α-diHCA, formed in a reaction catalysed by CYP7B1 (figure 1). 3β,7α-diHCA is then converted by HSD3B7 to 7αH,3O-CA, which then undergoes peroxisomal side-chain shortening to give the C_24_ acid 7α-hydroxy-3-oxochol-4-en-24-oic acid (7αH,3O-Δ^4^-BA); or 7αH,3O-CA may be first reduced in the A-ring by aldoketoreductase (AKR) 1D1 then by AKR1C4 prior to peroxisomal side-chain shortening to ultimately give chenodeoxycholic acid [5,6]. Alternatively, 3β,7α-diHCA itself can undergo side-chain shortening to give 3β,7α-dihydroxychol-5-en-24-oic acid (3β,7α-diH-Δ^5^-BA). Each of these pathways proceeds following 7α-hydroxylation of the core 3β-hydroxy-5-ene sterol. It is unknown whether CYP7B1 will accept 3-oxo-4-ene oxysterols and sterol-acids as substrates (figure 3), and whether 3O-CA will undergo side-chain shortening leading to 3-oxochol-4-en-24-oic acid (3O-Δ^4^-BA). Interrogation of the RIC at *m*/*z* 506.3377 ± 5 ppm corresponding to [^2^H_0_]GP-derivatized 3O-Δ^4^-BA indicates its presence in plasma from pregnant women but below the limit of quantification (0.2 ng ml^−1^, figure 5*c*,*d*; electronic supplementary material, figure S4B). In cord plasma, the concentration of 3O-Δ^4^-BA is still low (0.76 ± 0.47 ng ml^−1^) but about 23% that of 3β-hydroxychol-5-en-24-oic acid (3βH-Δ^5^-BA; figure 5*e*,*f*). In placenta, the concentration of 3O-Δ^4^-BA is also low at 1.26 ± 0.72 ng g^−1^ and about 16% that of 3βH-Δ^5^-BA (figure 5*g*,*h*). 3O-Δ^4^-BA was not observed in plasma from non-pregnant women (figure 5*a*).

The current data suggest that 3-oxo-4-ene sterol-acids may be formed in placenta even when they do not possess a 7α-hydroxy group. A pathway can be envisaged starting with 26-HC and involving the intermediates 26-HCO, 3O-CA and 3O-Δ^4^-BA (shown on a pale-yellow background in figure 3). Alternatively, like 26-HCO, 3O-CA and 3O-Δ^4^-BA could be formed directly from their 3β-hydroxy-5-ene equivalents by a HSD enzyme other than HSD3B7.

### 3.4. Placenta and umbilical cord blood contain novel intermediates in a pathway leading to progesterone

Progesterone is formed in steroidogenic tissue from cholesterol by mitochondrial CYP11A1 and HSD3B1/2 (figure 1) [21]; CYP11A1 and HSD3B1 are both enriched in placenta [9,19]. In the first step, cholesterol is converted to 22R-HC and then further to 20R,22R-diHC and onto pregnenolone all by CYP11A1 [22,23]. Pregnenolone is then converted to progesterone by HSD3B1 in placenta and by HSD3B2 in other steroidogenic tissues. The evidence presented above suggesting that 26-HC can be converted to 26-HCO in placenta raises the possibility that similar oxidation reactions may proceed with 22R-HC and 20R,22R-diHC as substrates leading to 22R-hydroxycholest-4-en-3-one (22R-HCO) and 20R,22R-dihydroxycholest-4-en-3-one (20R,22R-diHCO) products, providing an alternative route to progesterone involving the same enzymes as the conventional route but acting in a different order and avoiding pregnenolone (figure 3, see pathway on an orange background).

**Figure 6.**
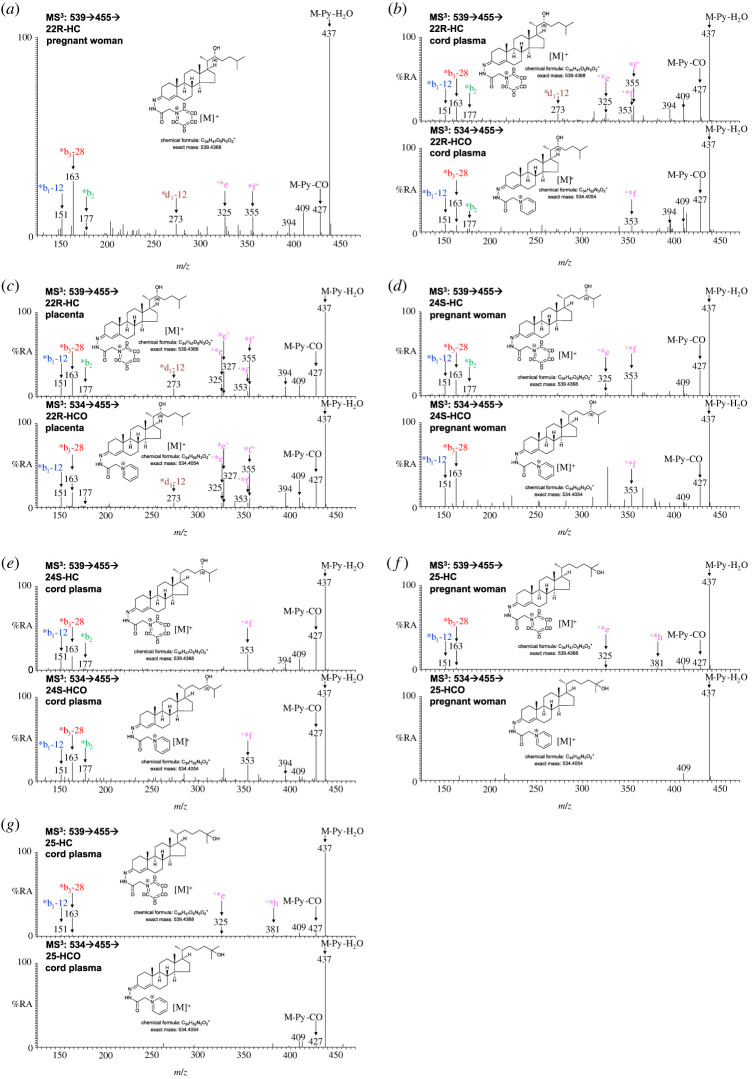
Hydroxycholestenones are present in pregnant women's and cord plasma and in placenta tissue. MS^3^ ([M]^+^ → [M-Py]^+^→) spectra of hydroxycholesterol (HC) plus hydroxycholestenone (HCO, where present) derivatized with [^2^H_5_]GP (upper panels) and hydroxycholestenones (if present) derivatized with [^2^H_0_]GP (lower panels). (*a*) 22R-HC is present in pregnant woman's and (*b*) cord plasma but 22R-HCO is at, or below, the detection limit in these plasmas, while it is present in (*c*) placenta. 24S-HCO is present in (*d*) pregnant woman's and (*e*) cord plasma. 25-HCO is at the detection limit in (*f*) pregnant woman's plasma and (*g*) cord plasma. See figure 8*d,e* for spectra of 24S-HCO and 25-HCO in placenta. Note the presence of the fragment ion at *m*/*z* 327 in the MS^3^ spectra of 24S-HCO from pregnant woman's and cord plasma (*d*,*e*) indicating the additional presence of minor amounts of 20S-HCO. See figure 4*c*,*e,g* for chromatograms from pregnant woman's and cord plasma, and placenta, respectively. There is some shift in retention time between samples which were analysed at different times and on different LC columns but of the same type. MS^3^ spectra can be compared to those of authentic standards [43].

22R-HCO was found to be present in only 3 out of the 14 cord plasma samples, and where its level was about 0.5 ng ml^−1^, for comparison 22R-HC was found in all samples at a level of 6.19 ± 3.01 ng ml^−1^ (figures 4*e* and 6*b*; electronic supplementary material, figure S2G) [44]. 22R-HCO was at or below the detection limit in plasma from pregnant and non-pregnant women (control) plasma, although 22R-HC was found in pregnant women's plasma (2.55 ± 1.18 ng ml^−1^; figures 4*a*,*c* and 6*a*) [44]. In placenta, the concentration of 22R-HCO was found to be 2.16 ± 0.38 ng g^−1^, 1% that of 22R-HC (figures 4*g* and 6*c*).

**Figure 7.**
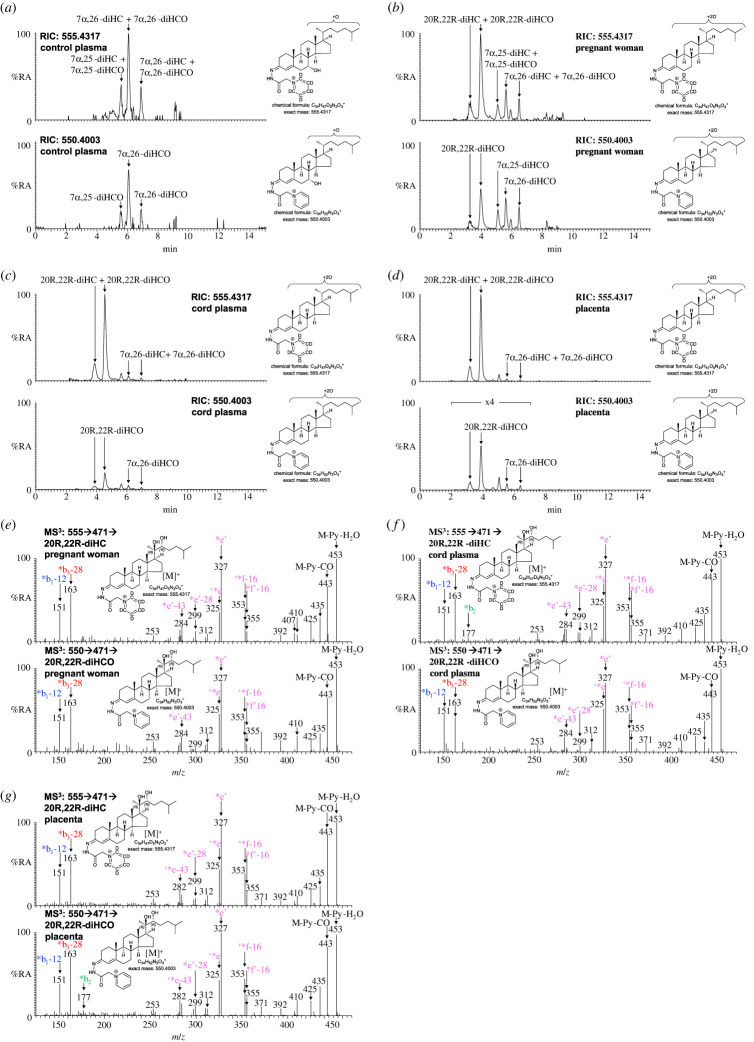
20R,22R-diHCO is present in pregnant women's and cord plasma and in placental tissue. RICs of 555.4317 ± 5 ppm corresponding to dihydroxycholesterols (diHC) plus dihydroxycholestenones (diHCO) including 20R,22R-diHC plus 20R,22R-diHCO when present, derivatized with [^2^H_5_]GP (upper panels) and 550.4003 ± 5 ppm corresponding to dihydroxycholestenones, including 20R,22R-diHCO if present, derivatized with [^2^H_0_]GP (lower panels). (*a*) Non-pregnant female (control) plasma, (*b*) pregnant woman's plasma, (*c*) cord plasma, and (*d*) placenta. Chromatograms in upper and lower panels are plotted on the same *y*-axis and magnified as indicated. MS^3^ ([M]^+^ → [M-Py]^+^→) spectra of 20R,22R-diHC plus 20R,22R-diHCO derivatized with [^2^H_5_]GP (upper panels) and 20R,22R-diHCO derivatized with [^2^H_0_]GP (lower panels) from (*e*) pregnant woman's plasma, (*f*) cord plasma, and (*g*) placenta. There is some shift in retention time between samples which were analysed at different times and on different LC columns but of the same type. MS^3^ spectra can be compared to those of authentic standards [43]. Further data can be found in electronic supplementary material, figure S5A–D.

20R,22R-diHCO was found in each of the cord plasma samples analysed. It was much more abundant than 22R-HCO, at a concentration of 12.86 ± 6.09 ng ml^−1^ corresponding to 26% that of 20R,22R-diHC (figure 7*c*,*f*; electronic supplementary material, figure S5C). In plasma from pregnant women 20R,22R-diHCO was present at a concentration of 6.04 ± 3.94 ng ml^−1^, 46% that of 20R,22R-diHC (figure 7*b*,*e*), although it, and 20R,22R-diHC, were absent from plasma from non-pregnant females (figure 7*a*). Both 20R,22R-diHCO and 20R,22R-diHC were present in placenta (figure 7*d*,*g*).

It is surprising that the ratio of 20R,22R-diHCO to 20R,22R-diHC is greater in plasma from pregnant women than cord plasma, but the fact that both these sterols are more abundant than their 20R-monohydroxy analogues raises the possibility of further metabolism of 20R,22R-diHCO by CYP11A1 to progesterone in the placenta or alternatively to intermediates analogous to a recently described pathway to bile acids [44].

### 3.5. 24S-Hydroxycholest-4-en-3-one is present in pregnant women's and cord plasma, 20S-hydroxycholest-4-en-3-one is present in placenta

In plasma from pregnant women, 24S-HC is the second most abundant side-chain monohydroxycholesterol after 26-HC (figures 4*c* and 6*d*; table 1). While 26-HCO was present in nine of the ten samples analysed, at about 3% of 26-HC, 24S-hydroxycholest-4-en-3-one (24S-HCO) could only be detected in three of the ten samples, giving a mean concentration of 0.04 ± 0.10 ng ml^−1^ which is less than 1% of the concentration of 24S-HC. 24S-HCO was below the limit of quantification in all five plasma samples from non-pregnant females (figure 4*a*). In cord plasma 24S-HCO was quantified in 7 of the 14 samples (figures 4*e* and 6*e*), leading to a mean concentration of 0.29 ± 0.39 ng ml^−1^, only 4% that of 24S-HC, compared with 26-HCO being 23% of 26-HC.

**Figure 8.**
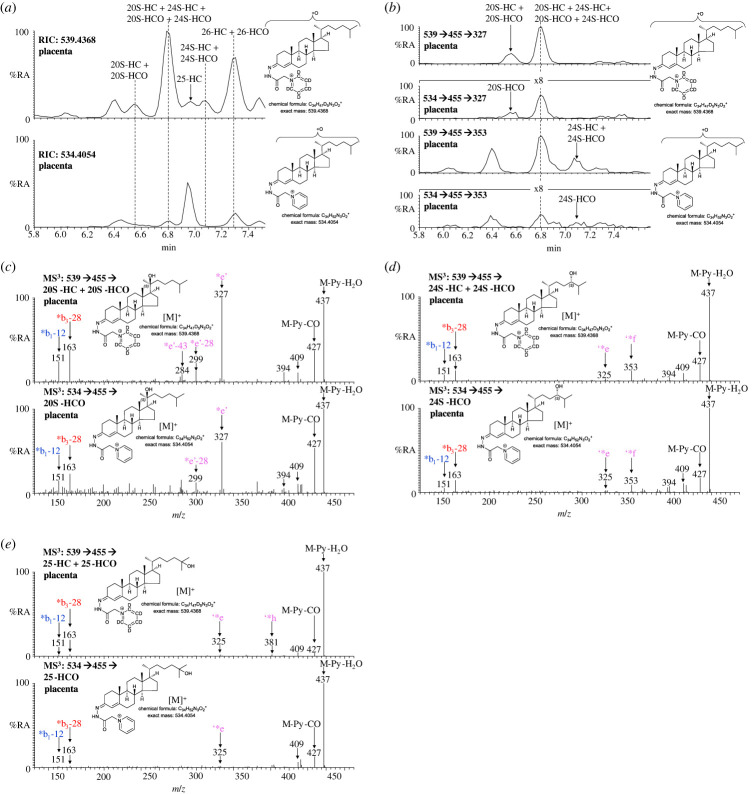
20S-HC, 20S-HCO, 24S-HC, 24S-HCO, 25-HC, 25-HCO, 26-HC and 26-HCO are present in placenta. (*a*) RICs of 539.4368 ± 5 ppm (upper panel) and 534.4054 ± 5 ppm (lower panel) over the elution time of 20S-HC, 20S-HCO, 24S-HC, 24S-HCO, 25-HC, 25-HCO, 26-HC and 26-HCO. The full-length chromatogram is presented in figure 4*g*. (*b*) MRM-like chromatograms targeting 20S-HC (top panel) and 20S-HCO (second panel), i.e. [M]^+^ → [M-Py]^+^ → 327, plotted on the same *y*-axis, and 24S-HC (third panel) and 24S-HCO (bottom panel), i.e. [M]^+^ → [M-Py] → 353, plotted on the same *y*-axis. MS^3^ ([M]^+^ → [M-Py]^+^→) spectra of (*c*) the first peaks of 20S-HC plus 20S-HCO (upper panel) and 20S-HCO (lower panel), (*d*) the second peaks of 24S-HC plus 24S-HCO (upper panel) and 24S-HCO (lower panel), and (*e*) 25-HC plus 25-HCO (upper panel) and 25-HCO (lower panel). Note the fragment ion at *m*/*z* 353 is characteristic of 24S-HC/24S-HCO while *m*/*z* 327 is characteristic of 20S-HC/20-HCO. Further data can be found in electronic supplementary material, figure S6.

20S-HC is not normally found in plasma, but it is reported to be present in rodent brain and human placenta [44–46]. Both 20S-HC and 24S-HC are found in placenta as are 20S-hydroxycholest-4-en-3-one (20S-HCO) and 24S-HCO (figures 4*g* and 8*a–d*; electronic supplementary material, figure S6). 20S-HC and 20S-HCO can be targeted by generating multiple reaction monitoring (MRM)-like chromatograms [M]^+^ → [M-Py]^+^ → 327 (figure 8*b*, first and second panels), while 24S-HC and 24S-HCO can be targeted by [M]^+^ → [M-Py] → 353 chromatograms (figure 8*b*, third and fourth panels). As is the case of most oxysterols, 20S-HC and 24S-HC and 20S-HCO and 24S-HCO each give twin peaks when derivatized with GP-hydrazine corresponding to *syn* and *anti* conformers about the hydrazone C=N double bond [43]. While the first peaks for 20S-HC and 20S-HCO are resolved from 24S-HC and 24S-HCO peaks, and the second peaks for 24S-HC and 24S-HCO are similarly resolved from the 20S-isomers, the second 20S-HC and 20S-HCO peaks co-elute with the first peaks of 24S-HC and 24S-HCO (figure 8*a*,*b*). This means that quantification in placenta is only approximate. As an estimation, 24S-HCO and 20S-HCO are present at about 5% of 24S-HC and 20S-HC, respectively.

### 3.6. Other 3-oxo-4-enes in plasma and placenta

Oxysterols with a 7α-hydroxy-3-oxo-4-ene structure are likely to be formed via the action of HSD3B7 on 3β,7α-dihydroxy-5-ene substrates (figure 1) [8]. Their identification and quantification in pregnant women's and cord plasma are presented elsewhere [44].

In both pregnant women's and cord plasma low levels of 25-hydroxycholest-4-en-3-one (25-HCO) are detected but below the limit of quantification (figure 4*c*,*e*; electronic supplementary material, figure S2F,G). 25-Hydroxycholesterol (25-HC) the likely precursor is, however, present at low levels in these two types of plasma at about 2.7 ng ml^−1^. 25-HC and 25-HCO are also found in placenta but were not quantified (figure 8*a*,*e*).

In previous studies, we have partially identified an oxysterol in plasma as either 3β,20-dihydroxycholest-5-en-22-one (3β,20-diHC-22O) or 3β,22-dihydroxycholest-5-en-24-one (3β,22-diHC-24O) but the identity was not confirmed due to the absence of authentic synthetic standards [43]. This oxysterol is present in the current samples generically named as 3β,x-diHC-yO (figure 2; electronic supplementary material, figure S7) and its concentration increases in the order non-pregnant women's plasma (10.59 ± 5.77 ng ml^−1^, figure 2*a*), pregnant women's plasma (21.72 ± 8.22 ng ml^−1^, figure 2*c*) and cord plasma (103.6 ± 44.51 ng ml^−1^, figure 2*e*). It is also present in placenta (figure 2*g*). Interestingly, the 3-oxo-4-ene version of this molecule (generically x-HC-3,y-diO), 20-hydroxycholest-4-ene-3,22-dione (20-HC-3,22-diO) or 22-hydroxycholest-4-ene-3,24-dione (22-HC-3,24-diO), shows the same trend in concentration, being below the limit of detection in non-pregnant female plasma, at 18.21 ± 6.91 ng ml^−1^ in plasma from pregnant women and 78.24 ± 30.85 ng ml^−1^ in cord plasma. These values correspond to less than 1%, 84% and 76% of the 3β-hydroxy-5-ene versions. 20-HC-3,22-diO or 22-HC-3,24-diO is also abundant in placenta. In the derivatization method employed in this study 24-oxo groups may be derived from natural 24,25-epoxy groups [43], which raises the possibility that this may also be the case here.

Another oxysterol previously found in plasma and partially identified, based on exact mass, retention time and MS^3^ data, is either 3β,25-dihydroxycholest-5-en-26-oic acid (3β,25-diHCA) or 3β,25,x-trihydroxycholest-5-en-y-one (3β,25,x-triHC-yO) [43]. In the present study, this compound is found at similar levels in plasma from non-pregnant (6.36 ± 3.05 ng ml^−1^, table 1; see electronic supplementary material, figure S8A–C) and pregnant women (6.34 ± 2.03 ng ml^−1^; electronic supplementary material, figure S8E–G) but is more abundant in cord plasma (13.02 ± 6.47 ng ml^−1^; see electronic supplementary material, figure S9A–C). As might be expected in light of the data presented above, the 3-oxo-4-ene version, i.e. either 25-hydroxy-3-oxocholest-4-en-26-oic acid (25H,3O-CA) or 25,x-dihydroxycholest-4-en-3,y-dione (25,x-diHC-3,y-diO), is absent from non-pregnant women's plasma (electronic supplementary material, figure S8A,B), but present at increasing concentration in pregnant women's plasma (4.29 ± 2.64 ng ml^−1^; electronic supplementary material, figure S8E–G) and cord plasma (12.12 ± 5.03 ng ml^−1^; electronic supplementary material, figure S9A–C), the latter two concentrations corresponding to 68% and 93% of their 3β-hydroxy-5-ene analogues. Both 3β,25-diHCA or 3β,25,x-triHC-y-O and 25H,3O-CA or 25,x-diHC-3,y-diO are present in placenta (electronic supplementary material, figure S9E–G).

A further partially identified pair of 3β-hydroxy-5-ene and 3-oxo-4-ene sterol-acids found in pregnant women's and cord plasma is 3β,x-dihydroxycholest-5-en-26-oic (3β,x-diHCA) and x-hydroxy-3-oxocholest-4-en-26-oic acid (xH,3O-CA; see electronic supplementary material, figures S8E,F,H and S9A,B,D). The extra hydroxy group designated by x is probably on the side-chain. Only the 3β-hydroxy-5-ene sterol-acid is found in non-pregnant females' plasma (see electronic supplementary material, figure S8A,B,D). The concentration of 3β,x-diHCA increases from non-pregnant women's plasma (4.68 ± 1.82 ng ml^−1^) to pregnant women's plasma (7.42 ± 2.95 ng ml^−1^) to cord plasma (34.00 ± 16.87 ng ml^−1^). The concentrations of xH,3O-CA in pregnant women's and cord plasma are 4.99 ± 2.25 ng ml^−1^ and 11.8 ± 4.60 ng ml^−1^, these values corresponding to 67% and 35%, respectively, of the equivalent 3β-hydroxy-5-ene sterols. Both 3β,x-diHCA and xH,3O-CA are also present in placenta (see electronic supplementary material, figure S9E,F,H). An alternative identification for this pair of 3β-hydroxy-5-ene and 3-oxo-4-ene sterols is 3β,x,y-trihydroxycholest-5-en-z-one (3β,x,y-triHC-zO) and x,y-dihydroxycholest-4-en-3,z-dione (x,y-diHC-3,z-diO). If this were the correct structure it is likely that the extra hydroxy groups designated x and y and the oxo group z are in the side-chain [43].

### 3.7. HSD3B1 converts 24S-HC and 20S-HC to 24S-HCO and 20S-HCO, respectively

HSD3B1 converts pregnenolone to progesterone (figure 1) and is highly expressed in placenta [9,19]. HSD3B7, the enzyme that converts 7α-hydroxysterols to their 3-oxo-4-ene equivalents does not use as substrates C_27_ sterols lacking a 7α-hydroxy group [8], and is not expressed in placenta [9]. Thus, HSD3B1 represents a plausible enzyme which is present in placenta that could have 3β-hydroxy-Δ^5^-C_27_-steroid oxidoreductase Δ^5^-isomerase activity and convert 3β-hydroxy-5-enes to their 3-oxo-4-ene equivalents. To test this hypothesis, the plasmid encoding human *HSD3B1* (pHSD3B1; see electronic supplementary material) was transfected into HEK 293 cells using JetOPTIMUS transfection reagent and the expression of protein confirmed by immunoblot analysis (electronic supplementary material, figure S10). The activity of HSD3B1 expressed in transfected HEK 293 cells towards 1 μM [^2^H_7_]24R/S-HC and [^2^H_7_]20S-HC was investigated in incubation buffer [47]. After 1 h of incubation, oxysterols were extracted from cell pellets (approx. 3 × 10^6^ cells) and subjected to EADSA and LC-MS(MS^3^). [^2^H_7_]22S-HCO and [^2^H_7_]7α-HC were included in the extraction solvent to act as internal standards to quantitatively monitor newly formed hydroxycholestenones and any spurious formation of 3-oxo-4-enes during sample preparation, respectively.

**Figure 9.**
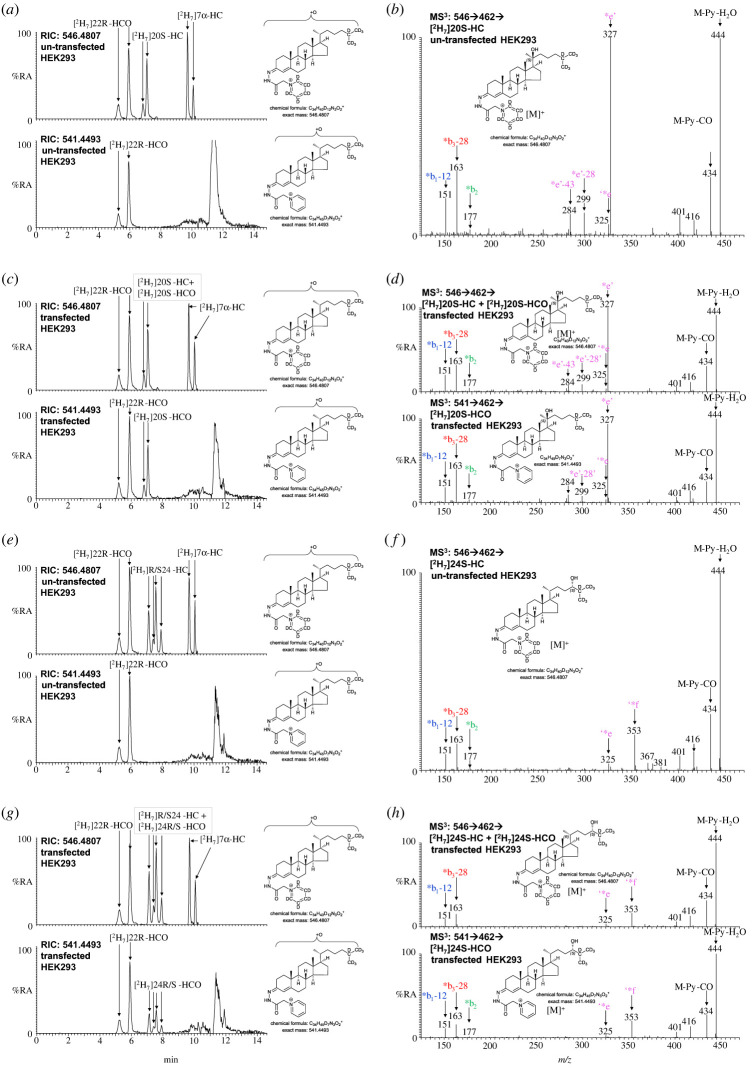
[^2^H_7_]20S-HC and [^2^H_7_]24R/S-HC are converted to [^2^H_7_]20S-HCO and [^2^H_7_]24R/S-HCO by HEK 293 cells transfected with HSD3B1. [^2^H_7_]20S-HC was incubated with un-transfected HEK 293 cells (*a,b*) or cells transfected with HSD3B1 (*c,d*). (*a,c*) RICs of *m*/*z* 546.4807 corresponding to GP-derivatized [^2^H_7_]20S-HC and [^2^H_7_]20S-HCO, if present, (upper panel) and *m*/*z* 541.4493 corresponding [^2^H_7_]20S-HCO (lower panel). [^2^H_7_]22R-HCO and [^2^H_7_]7α-HC were added in the extraction solvent to quantitatively monitor [^2^H_7_]20S-HC formation and any spurious oxidation at C-3. (*b,d*) MS^3^ ([M]^+^ → [M-Py]^+^→) spectra of [^2^H_7_]20S-HC plus [^2^H_7_]20S-HCO, if present (upper panel), and [^2^H_7_]20S-HCO, if present (lower panel). (*e,g*) RICs of *m*/*z* 546.4807 corresponding to GP-derivatized [^2^H_7_]24R/S-HC plus [^2^H_7_]24R/S-HCO, if present (upper panel), and *m*/*z* 541.4493 corresponding [^2^H_7_]24R/S-HCO (lower panel). (*f,h*) MS^3^ ([M]^+^ → [M-Py]^+^→) spectra of [^2^H_7_]24S-HC plus [^2^H_7_]24S-HCO, if present (upper panel), and [^2^H_7_]24S-HCO, if present (lower panel).

As is evident from figure 9*a*,*e* un-transfected cells do not convert [^2^H_7_]24R/S-HC or [^2^H_7_]20S-HC to their 3-ones. However, when HEK 293 cells were transfected with HSD3B1 and incubated for 1 h with 1 μM [^2^H_7_]20S-HC, about of 75% substrate was converted to [^2^H_7_]20S-HCO product (figure 9*c*,*d*). Similar results were obtained in incubations with [^2^H_7_]24R/S-HC but conversion to [^2^H_7_]24S-HCO was only about 35% (figure 9*g*,*h*).

## 4. Discussion

In the current study, we have made the surprising observation that oxysterols, including sterol-acids, with a 3-oxo-4-ene function but lacking a 7α-hydroxy group are present in plasma from pregnant women and from umbilical cord blood. The origin of these unexpected metabolites is likely to be the placenta based on their relative abundance in this tissue. In the analysis of adult plasma, oxysterols with a 3-oxo-4-ene function are nearly always found to be 7α-hydroxylated [38,41–43,48]. HSD3B7 is the C_27_ oxidoreductase Δ^5^-isomerase which converts the 3β-hydroxy-5-ene group in oxysterols possessing a 7α-hydroxy group to the 3-oxo-4-ene group (figure 1) [8]. HSD3B7 only uses C_27_ oxysterols with a 7α-hydroxy group as its substrates and does not convert pregnenolone to progesterone or oxidize other C_21_ steroids at C-3 [8]. Instead, this activity is the preserve of HSD3B1 and 3B2 [19–21,47]. HSD3B2 is primarily expressed in the adrenal gland, ovary and testis, while HSD3B1 is primarily localized to placenta and non-steroidogenic tissue [9,19]. In the placenta, HSD3B1 is reported to have both microsomal and mitochondrial locations, the latter being optimal for progesterone synthesis from pregnenolone, as CYP11A1 producing pregnenolone is also mitochondrial [49,50]. As HSD3B1 is a 3β-HSD Δ^5^ isomerase and is abundant in placenta, it represents a good candidate enzyme to catalyse the unexpected conversion of C_27_ 3β-hydroxy-5-ene oxysterols lacking a 7α-hydroxy group to their 3-oxo-4-ene analogues. We performed a proof-of-principle study where we incubated isotope-labelled versions of oxysterols (i.e. [^2^H_7_]24R/S-HC and [^2^H_7_]20S-HC), whose natural S-isomers are present in placenta, with HEK 293 cells transfected with *HSD3B1* and expressing HSD3B1. We selected these oxysterols as their natural-isotopic versions have previously been identified in placenta; although the former is not known to be synthesized in placenta, its presence is most likely from circulating maternal blood [44,45]. We found that both oxysterols were converted to their 3-oxo-4-ene forms. This preliminary study confirms that HSD3B1 has activity towards C_27_ oxysterols and is likely to be the enzyme converting oxysterols without a 7α-hydroxy group from their 3β-hydroxy-5-ene to 3-oxo-4-ene form.

When unexpected oxysterols are identified, it is wise to be wary of *ex vivo* autoxidation leading to artefact formation. We were able to confirm that this was not the case here by the inclusion of isotope-labelled standards in the extraction solvent and by noting an absence of isotope-labelled oxysterols with a changed structure. Another potential stage for autoxidation is during sample storage. However, the most labile positions in cholesterol are at C-4 and C-7 allylic to the Δ^5^ double bond [51–54], and it is difficult to conceive a simple free radical mechanism for the transition of a 3β-hydrox-5-ene group to a 3-oxo-4-ene and a more plausible route is therefore through enzymatic oxidation. Bacterial cholesterol oxidase can convert the 3β-hydroxy-5-ene group to the 3-oxo-4-ene [55], and in our analytical method we perform such a reaction (electronic supplementary material, figure S1); however, we detect the unexpected 3-oxo-4-ene oxysterols in the absence of added cholesterol oxidase. Bacteria in the uterus could provide an alternative source of cholesterol oxidase activity and interestingly mycobacteria can express a 3β-HSD which will convert 25-HC to 25-HCO [56]. An alternative source of cholesterol oxidase activity has been found to be a complex of Cu^2+^ and amyloid β-peptide [57], and amyloid beta precursor protein does have some expression in placenta as does beta secretase 1 and the gamma secretase components presenilin-1, nicastrin, anterior pharynx-defective 1 and presenilin enhancer 2 [9], required for amyloid β-peptide formation. Copper is also present in placenta at a level of about 1 µg g^−1^ [58]. However, our finding that cells expressing human HSD3B1 will convert the 3β-hydroxy-5-ene function to the 3-oxo-4-ene, at least in [^2^H_7_]24R/S-HC and [^2^H_7_]20S-HC, strongly favours the conclusion that the unexpected oxysterols with a 3-oxo-4-ene group are formed endogenously in placenta by this enzyme.

**Figure 10.**
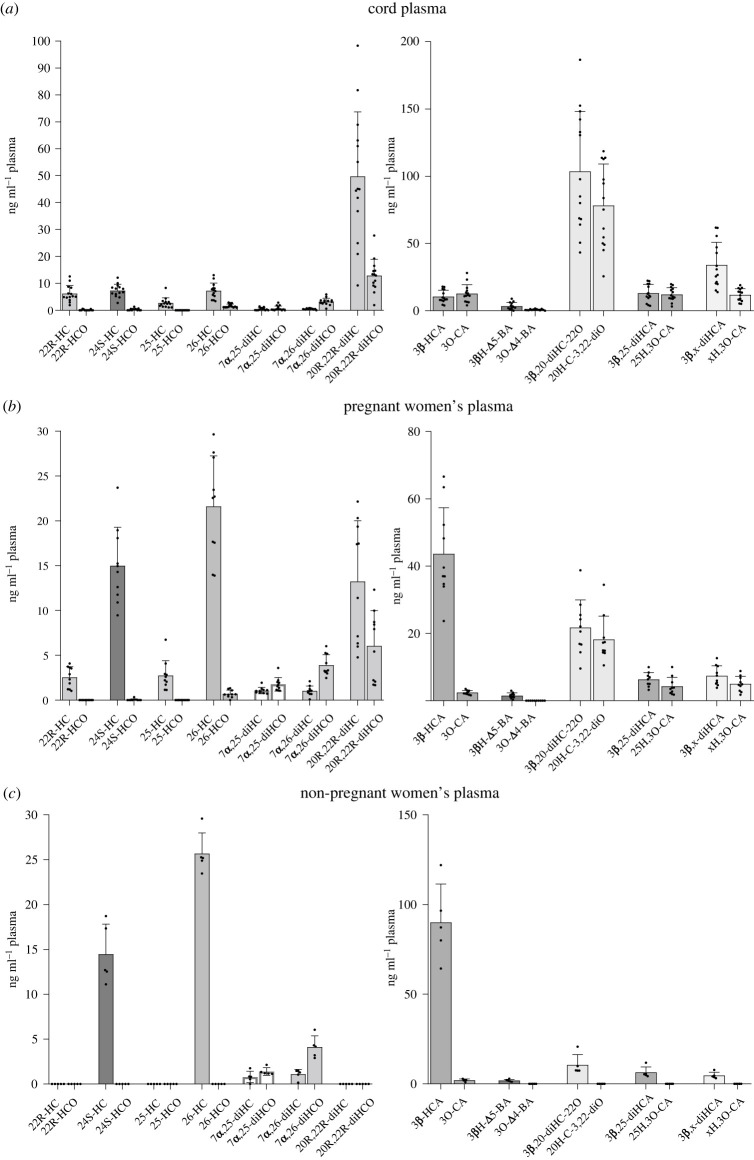
Concentrations of oxysterols in plasma from (*a*) the umbilical cord, (*b*) pregnant women and (*c*) non-pregnant women (controls).

What might be the biological benefit of placental HSD3B1 having activity towards C_27_ sterols? As cord blood is the blood remaining in the umbilical cord and placenta following birth its composition can give clues to the biology of oxysterols with the 3-oxo-4-ene function (figure 10). The oxysterol composition of cord plasma indicates two unusual pathways of metabolism. In the first we see a pathway of 26-HCO, 3O-CA and 3O-Δ^4^-BA providing a route towards bile acids as shown on the pale-yellow background in figure 3. 26-HCO may be formed via HSD3B1 oxidation of 26-HC and then metabolized to 3O-CA by CYP27A1. CYP27A1 has been shown to be active towards sterols with a 3-oxo-4-ene structure [59]. Peroxisomal side-chain shortening will then lead to 3O-Δ^4^-BA. 26-HC is an LXR ligand [60] and also a selective oestrogen receptor modulator [61], so oxidation at C-3 could provide a route to deactivate the ligand. Two alternative routes of deactivation of 26-HC are (i) CYP27A1 mediated oxidation to 3β-HCA, although 3β-HCA is itself an LXR ligand [62,63], and (ii) 7α-hydroxylation by CYP7B1 to 7α,26-diHC, but 7α,26-diHC is also biologically active as a chemoattractant of GPR183 expressing immune cells [13]. Interestingly, CYP27A1 is a mitochondrial enzyme [4], and HSD3B1 can also be located in this organelle [49], thus HSD3B1 oxidation of 3β-HCA to 3O-CA could act as a route for the former acid's deactivation. Conversion of the 3β-hydroxy-5-ene function in 7α,26-diHC by HSD3B7 to the 3-oxo-4-ene in 7α,26-dihydroxycholest-4-en-3-one (7α,26-diHCO) is an established route of its deactivation [13]. In each of the plasma samples 7α,26-diHC is only a minor oxysterol, 7α,26-diHCO being three to seven times more abundant (table 1 and figure 10).

The second unusual metabolic pathway revealed by the analysis of cord plasma also involves oxysterols with a 3-oxo-4-ene structure and encompasses 20R-HC, 20R,22R-diHC, 20R-HCO and 20R,22R-diHCO, presumably leading to progesterone by side-chain cleavage of 20R,22R-diHCO (figure 3, orange background). This pathway may again be a route to deactivate LXR ligands, in this case 22R-HC and 20R,22R-diHC [24], or a route to progesterone avoiding pregnenolone, although using the same enzymes CYP11A1 and HSD3B1 as in the conventional pathway but in a different order. The introduction to this pathway may be through HSD3B1 oxidation of either 22R-HC or 20R,22R-diHC, CYP11A1 being a 22- and 20-hydroxylase and also the side-chain shortening enzyme [22]. It is perhaps significant that like CYP27A1, CYP11A1 is a mitochondrial enzyme, while HSD3B1 also has a mitochondrial location. Certainly, by studying figure 10 it is evident that 3-oxo-4-ene metabolites are most prevalent in compounds with 20- or 22-hydroxylation (introduced by CYP11A1) or 26-hydroxylation or carboxylation (introduced by CYP27A1). Extrapolating this evidence to 20S-HC and 20S-HCO suggests that the enzyme generating 20S-HC from cholesterol is mitochondrial, perhaps CYP11A1.

20S-HC like many other side-chain oxysterols inhibits the processing of SREBP-2 [27,28] and is an LXR ligand [24,26]. It is also a reported agonist towards another nuclear receptor, retinoic acid receptor-related orphan receptor *γ* (ROR*γ*) [64], an activator of the Hh signalling pathway by binding to SMO [29], and a ligand to the *σ*2 receptor, encoded by *TMEM97* [30]. Interestingly, *TMEM97* is overexpressed in proliferating tumours and believed to be involved in cholesterol homeostasis [30]. 20S-HC has previously been reported in human placenta and rodent brain [44–46]. The metabolism of 20S-HC has not been studied extensively [1]; however, it can bind to the active site of CYP11A1 [65], and has been shown to be converted to pregnenolone [66], although this reaction is not considered as a major route to pregnenolone [1]. Besides inhibiting the processing of SREBP-2 [28], 20S-HC will also repress the activity of hydroxymethylglutaryl (HMG)-CoA reductase [67], as will other side-chain oxysterols [68]. Although produced primarily in brain [69,70], 24S-HC is abundant in the circulation [43] (table 1 and figure 10) and has a route from mother to fetus via the placenta. Like 20S-HC, 24S-HC is a ligand to LXRs and inhibits the processing of SREBP-2 [25–27], but unlike 20S-HC is an inverse agonist towards ROR*γ* [71]. Of these myriads of activities, few have been ascribed to the 3-oxo-4-ene sterols, suggesting once more that HSD3B1 can provide a role in deactivating oxysterols.

It should be pointed out that very few studies have been performed on oxysterols with 3-oxo-4-ene function but devoid of a 7α-hydroxy group [1,14,63,67,72]. This is probably because they are seldom identified *in vivo*. Interestingly, the trio of metabolites 26-HCO, 3O-CA and 3O-Δ^4^-BA provide an exception in that like 25-HC and 26-HC they will each suppress the activity of HMG-CoA reductase in human fibroblasts [72]. These cells will convert 26-HCO to 3O-CA [72], in agreement with other studies showing 3-oxo-4-ene sterols are substrates for CYP27A1 [59].

In summary, the placenta facilitates exchange of metabolites between the fetus and mother. It is also an endocrine organ producing hormones that regulate maternal and fetal physiology. The umbilical cord connects the placenta to the fetus and its blood content can be sampled after birth as cord blood representing the fetal blood content of the placenta. By analysing plasma from pregnant women, cord blood and placental tissue along with plasma from non-pregnant women we provide evidence for the conversion of oxysterols with a 3β-hydroxy-5-ene structure to 3-oxo-4-ene analogues in placenta. Based on a proof-of-principle study this activity is likely to be catalysed by HSD3B1. We speculate that these unexpected reactions provide a mechanism to regulate biologically active oxysterols and deserves further study.

## Data Availability

All mass spectrometry raw data have been submitted to the Open Science Framework. Mass spectrometry data: OSF https://doi.org/10.17605/OSF.IO/EGNCZ [73]. The data are provided in electronic supplementary material [74].

## References

[1] Schroepfer Jr GJ. 2000 Oxysterols: modulators of cholesterol metabolism and other processes. Physiol. Rev. **80**, 361-554. (10.1152/physrev.2000.80.1.361) 10617772

[2] Setchell KD et al. 1998 Identification of a new inborn error in bile acid synthesis: mutation of the oxysterol 7alpha-hydroxylase gene causes severe neonatal liver disease. J. Clin. Invest. **102**, 1690-1703. (10.1172/JCI2962) 9802883 PMC509117

[3] Li-Hawkins J, Lund EG, Bronson AD, Russell DW. 2000 Expression cloning of an oxysterol 7α-hydroxylase selective for 24-hydroxycholesterol. J. Biol. Chem. **275**, 16 543-16 549. (10.1074/jbc.M001810200) 10748047

[4] Cali JJ, Russell DW. 1991 Characterization of human sterol 27-hydroxylase. A mitochondrial cytochrome P-450 that catalyzes multiple oxidation reaction in bile acid biosynthesis. J. Biol. Chem. **266**, 7774-7778. (10.1016/S0021-9258(20)89517-9) 1708392

[5] Russell DW. 2003 The enzymes, regulation, and genetics of bile acid synthesis. Annu. Rev. Biochem. **72**, 137-174. (10.1146/annurev.biochem.72.121801.161712) 12543708

[6] Griffiths WJ, Wang Y. 2020 Oxysterols as lipid mediators: their biosynthetic genes, enzymes and metabolites. Prostaglandins Other Lipid Mediat. **147**, 106381. (10.1016/j.prostaglandins.2019.106381) 31698146 PMC7081179

[7] Clayton PT, Leonard JV, Lawson AM, Setchell KD, Andersson S, Egestad B, Sjovall J. 1987 Familial giant cell hepatitis associated with synthesis of 3 beta, 7 alpha-dihydroxy- and 3 beta,7 alpha, 12 alpha-trihydroxy-5-cholenoic acids. J. Clin. Invest. **79**, 1031-1038. (10.1172/JCI112915) 3470305 PMC424280

[8] Schwarz M, Wright AC, Davis DL, Nazer H, Björkhem I, Russell DW. 2000 The bile acid synthetic gene 3beta-hydroxy-Delta(5)-C(27)-steroid oxidoreductase is mutated in progressive intrahepatic cholestasis. J. Clin. Invest. **106**, 1175-1184. (10.1172/JCI10902) 11067870 PMC301421

[9] Uhlen M et al. 2015 Proteomics. Tissue-based map of the human proteome. Science **347**, 1260419. (10.1126/science.1260419) 25613900

[10] Griffiths WJ, Sjovall J. 2010 Bile acids: analysis in biological fluids and tissues. J. Lipid Res. **51**, 23-41. (10.1194/jlr.R001941) 20008121 PMC2789783

[11] Hofmann AF, Hagey LR. 2014 Key discoveries in bile acid chemistry and biology and their clinical applications: history of the last eight decades. J. Lipid Res. **55**, 1553-1595. (10.1194/jlr.R049437) 24838141 PMC4109754

[12] Cohen JC, Cali JJ, Jelinek DF, Mehrabian M, Sparkes RS, Lusis AJ, Russell DW, Hobbs HH. 1992 Cloning of the human cholesterol 7 alpha-hydroxylase gene (CYP7) and localization to chromosome 8q11-q12. Genomics **14**, 153-161. (10.1016/S0888-7543(05)80298-8) 1358792

[13] Hannedouche S et al. 2011 Oxysterols direct immune cell migration via EBI2. Nature **475**, 524-527. (10.1038/nature10280) 21796212 PMC4297623

[14] Theofilopoulos S et al. 2014 Cholestenoic acids regulate motor neuron survival via liver X receptors. J. Clin. Invest. **124**, 4829-4842. (10.1172/JCI68506) 25271621 PMC4347238

[15] Fakheri RJ, Javitt NB. 2012 27-Hydroxycholesterol, does it exist? On the nomenclature and stereochemistry of 26-hydroxylated sterols. Steroids **77**, 575-577. (10.1016/j.steroids.2012.02.006) 22366074

[16] Subramaniam P, Clayton PT, Portmann BC, Mieli-Vergani G, Hadzić N. 2010 Variable clinical spectrum of the most common inborn error of bile acid metabolism—3beta-hydroxy-Delta 5-C27-steroid dehydrogenase deficiency. J. Pediatr. Gastroenterol. Nutr. **50**, 61-66. (10.1097/MPG.0b013e3181b47b34) 19915491

[17] Kavanagh KL, Jörnvall H, Persson B, Oppermann U. 2008 Medium- and short-chain dehydrogenase/reductase gene and protein families. Cell. Mol. Life Sci. **65**, 3895. (10.1007/s00018-008-8588-y) 19011750 PMC2792337

[18] Persson B et al. 2009 The SDR (short-chain dehydrogenase/reductase and related enzymes) nomenclature initiative. Chem. Biol. Interact. **178**, 94-98. (10.1016/j.cbi.2008.10.040) 19027726 PMC2896744

[19] Simard J, Ricketts ML, Gingras S, Soucy P, Feltus FA, Melner MH. 2005 Molecular biology of the 3β-hydroxysteroid dehydrogenase/Δ^5^-Δ^4^ isomerase gene family. Endocr. Rev. **26**, 525-582. (10.1210/er.2002-0050) 15632317

[20] Morel Y, Mébarki F, Rhéaume E, Sanchez R, Forest MG, Simard J. 1997 Structure-function relationships of 3β-hydroxysteroid dehydrogenase: contribution made by the molecular genetics of 3β-hydroxysteroid dehydrogenase deficiency. Steroids **62**, 176-184. (10.1016/S0039-128X(96)00178-X) 9029734

[21] Shackleton CH. 2012 Role of a disordered steroid metabolome in the elucidation of sterol and steroid biosynthesis. Lipids **47**, 1-12. (10.1007/s11745-011-3605-6) 21874273 PMC3564490

[22] Mast N, Annalora AJ, Lodowski DT, Palczewski K, Stout CD, Pikuleva IA. 2011 Structural basis for three-step sequential catalysis by the cholesterol side chain cleavage enzyme CYP11A1. J. Biol. Chem. **286**, 5607-5613. (10.1074/jbc.M110.188433) 21159775 PMC3037674

[23] Yoshimoto FK, Jung IJ, Goyal S, Gonzalez E, Guengerich FP. 2016 Isotope-labeling studies support the electrophilic compound I iron active species, FeO^3+^, for the carbon-carbon bond cleavage reaction of the cholesterol side-chain cleavage enzyme, cytochrome P450 11A1. J. Am. Chem. Soc. **138**, 12 124-12 141. (10.1021/jacs.6b04437) PMC5289895 27571509

[24] Forman BM, Ruan B, Chen J, Schroepfer GJ, Evans RM. 1997 The orphan nuclear receptor LXRα is positively and negatively regulated by distinct products of mevalonate metabolism. Proc. Natl Acad. Sci. **94**, 10 588-10 593. (10.1073/pnas.94.20.10588) PMC23411 9380679

[25] Janowski BA, Grogan MJ, Jones SA, Wisely GB, Kliewer SA, Corey EJ, Mangelsdorf DJ. 1999 Structural requirements of ligands for the oxysterol liver X receptors LXRα and LXRβ. Proc. Natl Acad. Sci. USA **96**, 266-271. (10.1073/pnas.96.1.266) 9874807 PMC15128

[26] Lehmann JM et al. 1997 Activation of the nuclear receptor LXR by oxysterols defines a new hormone response pathway. J. Biol. Chem. **272**, 3137-3140. (10.1074/jbc.272.6.3137) 9013544

[27] Radhakrishnan A, Ikeda Y, Kwon HJ, Brown MS, Goldstein JL. 2007 Sterol-regulated transport of SREBPs from endoplasmic reticulum to Golgi: oxysterols block transport by binding to Insig. Proc. Natl Acad. Sci. USA **104**, 6511-6518. (10.1073/pnas.0700899104) 17428920 PMC1851665

[28] Abrams ME et al. 2020 Oxysterols provide innate immunity to bacterial infection by mobilizing cell surface accessible cholesterol. Nat. Microbiol. **5**, 929-942. (10.1038/s41564-020-0701-5) 32284563 PMC7442315

[29] Nachtergaele S, Mydock LK, Krishnan K, Rammohan J, Schlesinger PH, Covey DF, Rohatgi R. 2012 Oxysterols are allosteric activators of the oncoprotein Smoothened. Nat. Chem. Biol. **8**, 211-220. (10.1038/nchembio.765) 22231273 PMC3262054

[30] Cheng Y-S et al. 2021 A proteome-wide map of 20(S)-hydroxycholesterol interactors in cell membranes. Nat. Chem. Biol. **17**, 1271-1280. (10.1038/s41589-021-00907-2) 34799735 PMC8607797

[31] Hoflinger P et al. 2021 Metabolic profiling in serum, cerebrospinal fluid, and brain of patients with cerebrotendinous xanthomatosis. J. Lipid Res. **62**, 100078. (10.1016/j.jlr.2021.100078) 33891937 PMC8135047

[32] Lutjohann D et al. 2018 International descriptive and interventional survey for oxycholesterol determination by gas- and liquid-chromatographic methods. Biochimie **153**, 26-32. (10.1016/j.biochi.2018.07.016) 30063945

[33] Schott HF, Lutjohann D. 2015 Validation of an isotope dilution gas chromatography-mass spectrometry method for combined analysis of oxysterols and oxyphytosterols in serum samples. Steroids **99**, 139-150. (10.1016/j.steroids.2015.02.006) 25701095

[34] Stiles AR, Kozlitina J, Thompson BM, McDonald JG, King KS, Russell DW. 2014 Genetic, anatomic, and clinical determinants of human serum sterol and vitamin D levels. Proc. Natl Acad. Sci. USA **111**, E4006-E4014. (10.1073/pnas.1413561111) 25201972 PMC4183318

[35] Roberg-Larsen H et al. 2014 Highly automated nano-LC/MS-based approach for thousand cell-scale quantification of side chain-hydroxylated oxysterols. J. Lipid Res. **55**, 1531-1536. (10.1194/jlr.D048801) 24792927 PMC4076067

[36] Honda A, Yamashita K, Hara T, Ikegami T, Miyazaki T, Shirai M, Xu G, Numazawa M, Matsuzaki Y. 2009 Highly sensitive quantification of key regulatory oxysterols in biological samples by LC-ESI-MS/MS. J. Lipid Res. **50**, 350-357. (10.1194/jlr.D800040-JLR200) 18815436

[37] McDonald JG, Smith DD, Stiles AR, Russell DW. 2012 A comprehensive method for extraction and quantitative analysis of sterols and secosteroids from human plasma. J. Lipid Res. **53**, 1399-1409. (10.1194/jlr.D022285) 22517925 PMC3371252

[38] Meaney S et al. 2007 Novel route for elimination of brain oxysterols across the blood-brain barrier: conversion into 7α-hydroxy-3-oxo-4-cholestenoic acid. J. Lipid Res. **48**, 944-951. (10.1194/jlr.M600529-JLR200) 17251592

[39] Zhang J, Xue Y, Jondal M, Sjovall J. 1997 7α-Hydroxylation and 3-dehydrogenation abolish the ability of 25-hydroxycholesterol and 27-hydroxycholesterol to induce apoptosis in thymocytes. Eur. J. Biochem. **247**, 129-135. (10.1111/j.1432-1033.1997.00129.x) 9249018

[40] Crick PJ et al. 2015 Quantitative charge-tags for sterol and oxysterol analysis. Clin. Chem. **61**, 400-411. (10.1373/clinchem.2014.231332) 25512642

[41] Crick PJ, Griffiths WJ, Zhang J, Beibel M, Abdel-Khalik J, Kuhle J, Sailer AW, Wang Y. 2017 Reduced plasma levels of 25-hydroxycholesterol and increased cerebrospinal fluid levels of bile acid precursors in multiple sclerosis patients. Mol. Neurobiol. **54**, 8009-8020. (10.1007/s12035-016-0281-9) 27878760 PMC5684259

[42] Abdel-Khalik J et al. 2017 Defective cholesterol metabolism in amyotrophic lateral sclerosis. J. Lipid Res. **58**, 267-278. (10.1194/jlr.P071639) 27811233 PMC5234729

[43] Yutuc E et al. 2021 Deep mining of oxysterols and cholestenoic acids in human plasma and cerebrospinal fluid: quantification using isotope dilution mass spectrometry. Anal. Chim. Acta **1154**, 338259. (10.1016/j.aca.2021.338259) 33736801 PMC7988461

[44] Dickson AL, Yutuc E, Thornton CA, Wang Y, Griffiths WJ. 2022 Identification of unusual oxysterols biosynthesised in human pregnancy by charge-tagging and liquid chromatography–mass spectrometry. Front. Endocrinol. (Lausanne) **13**, 1031013. (10.3389/fendo.2022.1031013) 36440193 PMC9685423

[45] Lin YY, Welch M, Lieberman S. 2003 The detection of 20S-hydroxycholesterol in extracts of rat brains and human placenta by a gas chromatograph/mass spectrometry technique. J. Steroid Biochem. Mol. Biol. **85**, 57-61. (10.1016/S0960-0760(03)00137-7) 12798357

[46] Yutuc E et al. 2020 Localization of sterols and oxysterols in mouse brain reveals distinct spatial cholesterol metabolism. Proc. Natl Acad. Sci. USA **117**, 5749-5760. (10.1073/pnas.1917421117) 32132201 PMC7084107

[47] Lachance Y, Luu-The V, Labrie C, Simard J, Dumont M, de Launoit Y, Guérin S, Leblanc G, Labrie F. 1990 Characterization of human 3β-hydroxysteroid dehydrogenase/Δ^5^-Δ^4^-isomerase gene and its expression in mammalian cells. J. Biol. Chem. **265**, 20 469-20 475. (10.1016/S0021-9258(17)30528-8) 2243100

[48] Axelson M, Mork B, Sjovall J. 1988 Occurrence of 3β-hydroxy-5-cholestenoic acid, 3β,7α-dihydroxy-5-cholestenoic acid, and 7α-hydroxy-3-oxo-4-cholestenoic acid as normal constituents in human blood. J. Lipid Res. **29**, 629-641. (10.1016/S0022-2275(20)38509-6) 3411238

[49] Thomas JL, Myers RP, Strickler RC. 1989 Human placental 3β-hydroxy-5-ene-steroid dehydrogenase and steroid 5→4-ene-isomerase: purification from mitochondria and kinetic profiles, biophysical characterization of the purified mitochondrial and microsomal enzymes. J. Steroid Biochem. **33**, 209-217. (10.1016/0022-4731(89)90296-3) 2770297

[50] Chapman JC, Polanco JR, Min S, Michael SD. 2005 Mitochondrial 3 beta-hydroxysteroid dehydrogenase (HSD) is essential for the synthesis of progesterone by corpora lutea: an hypothesis. Reprod. Biol. Endocrinol. **3**, 11. (10.1186/1477-7827-3-11) 15804366 PMC1087504

[51] Murphy RC, Johnson KM. 2008 Cholesterol, reactive oxygen species, and the formation of biologically active mediators. J. Biol. Chem. **283**, 15 521-15 525. (10.1074/jbc.R700049200) PMC2414298 18285330

[52] Iuliano L. 2011 Pathways of cholesterol oxidation via non-enzymatic mechanisms. Chem. Phys. Lipids **164**, 457-468. (10.1016/j.chemphyslip.2011.06.006) 21703250

[53] Yin H, Xu L, Porter NA. 2011 Free radical lipid peroxidation: mechanisms and analysis. Chem. Rev. **111**, 5944-5972. (10.1021/cr200084z) 21861450

[54] Xu L, Porter NA. 2015 Free radical oxidation of cholesterol and its precursors: implications in cholesterol biosynthesis disorders. Free Radic. Res. **49**, 835-849. (10.3109/10715762.2014.985219) 25381800 PMC4461549

[55] MacLachlan J, Wotherspoon AT, Ansell RO, Brooks CJ. 2000 Cholesterol oxidase: sources, physical properties and analytical applications. J. Steroid Biochem. Mol. Biol. **72**, 169-195. (10.1016/S0960-0760(00)00044-3) 10822008

[56] Varaksa T et al. 2021 Metabolic fate of human immunoactive sterols in *Mycobacterium tuberculosis*. J. Mol. Biol. **433**, 166763. (10.1016/j.jmb.2020.166763) 33359098

[57] Puglielli L et al. 2005 Alzheimer disease beta-amyloid activity mimics cholesterol oxidase. J. Clin. Invest. **115**, 2556-2563. (10.1172/JCI23610) 16127459 PMC1190368

[58] de Moraes ML, de Faria Barbosa R, Santo RE, da Silva Santos F, de Almeida LB, de Jesus EFO, de Carvalho Sardinha FL, das Graças Tavares do Carmo M. 2011 Distribution of calcium, iron, copper, and zinc in two portions of placenta of teenager and adult women. Biol. Trace Element Res. **143**, 1271-1281. (10.1007/s12011-011-8963-7) 21267672

[59] Norlin M, von Bahr S, Bjorkhem I, Wikvall K. 2003 On the substrate specificity of human CYP27A1: implications for bile acid and cholestanol formation. J. Lipid Res. **44**, 1515-1522. (10.1194/jlr.M300047-JLR200) 12777473

[60] Fu X, Menke JG, Chen Y, Zhou G, MacNaul KL, Wright SD, Sparrow CP, Lund EG. 2001 27-Hydroxycholesterol is an endogenous ligand for liver X receptor in cholesterol-loaded cells. J. Biol. Chem. **276**, 38 378-38 387. (10.1074/jbc.M105805200) 11504730

[61] Umetani M, Domoto H, Gormley AK, Yuhanna IS, Cummins CL, Javitt NB, Korach KS, Shaul PW, Mangelsdorf DJ. 2007 27-Hydroxycholesterol is an endogenous SERM that inhibits the cardiovascular effects of estrogen. Nat. Med. **13**, 1185-1192. (10.1038/nm1641) 17873880

[62] Song C, Liao S. 2000 Cholestenoic acid is a naturally occurring ligand for liver X receptor alpha. Endocrinology **141**, 4180-4184. (10.1210/endo.141.11.7772) 11089551

[63] Ogundare M, Theofilopoulos S, Lockhart A, Hall LJ, Arenas E, Sjovall J, Brenton AG, Wang Y, Griffiths WJ. 2010 Cerebrospinal fluid steroidomics: are bioactive bile acids present in brain? J. Biol. Chem. **285**, 4666-4679. (10.1074/jbc.M109.086678) 19996111 PMC2836072

[64] Jin L, Martynowski D, Zheng S, Wada T, Xie W, Li Y. 2010 Structural basis for hydroxycholesterols as natural ligands of orphan nuclear receptor RORγ. Mol. Endocrinol. **24**, 923-929. (10.1210/me.2009-0507) 20203100 PMC2870936

[65] Lambeth JD, Kitchen SE, Farooqui AA, Tuckey R, Kamin H. 1982 Cytochrome P-450scc-substrate interactions. Studies of binding and catalytic activity using hydroxycholesterols. J. Biol. Chem. **257**, 1876-1884. (10.1016/S0021-9258(19)68119-6) 7056749

[66] Roberts KD, Bandy L, Lieberman S. 1969 The occurrence and metabolism of 20α-hydroxycholesterol in bovine adrenal preparations. Biochemistry **8**, 1259-1270. (10.1021/bi00831a061) 4889094

[67] Taylor FR, Saucier SE, Shown EP, Parish EJ, Kandutsch AA. 1984 Correlation between oxysterol binding to a cytosolic binding protein and potency in the repression of hydroxymethylglutaryl coenzyme A reductase. J. Biol. Chem. **259**, 12 382-12 387. (10.1016/S0021-9258(18)90757-X) 6490619

[68] Song BL, Javitt NB, DeBose-Boyd RA. 2005 Insig-mediated degradation of HMG CoA reductase stimulated by lanosterol, an intermediate in the synthesis of cholesterol. Cell Metab. **1**, 179-189. (10.1016/j.cmet.2005.01.001) 16054061

[69] Lund EG, Xie C, Kotti T, Turley SD, Dietschy JM, Russell DW. 2003 Knockout of the cholesterol 24-hydroxylase gene in mice reveals a brain-specific mechanism of cholesterol turnover. J. Biol. Chem. **278**, 22 980-22 988. (10.1074/jbc.M303415200) 12686551

[70] Bjorkhem I. 2007 Rediscovery of cerebrosterol. Lipids **42**, 5-14. (10.1007/s11745-006-1003-2) 17393206

[71] Wang Y, Kumar N, Crumbley C, Griffin PR, Burris TP. 2010 A second class of nuclear receptors for oxysterols: regulation of RORα and RORγ activity by 24S-hydroxycholesterol (cerebrosterol). Biochim. Biophys. Acta **1801**, 917-923. (10.1016/j.bbalip.2010.02.012) 20211758 PMC2886165

[72] Axelson M, Larsson O, Zhang J, Shoda J, Sjovall J. 1995 Structural specificity in the suppression of HMG-CoA reductase in human fibroblasts by intermediates in bile acid biosynthesis. J. Lipid Res. **36**, 290-298. (10.1016/S0022-2275(20)39905-3) 7751816

[73] Dickson A, Yutuc E, Thornton CA, Dunford JE, Oppermann U, Wang Y, Griffiths WJ. 2023 Mass spectrometry data: OSF. (10.17605/OSF.IO/EGNCZ) PMC10154939 37132223

[74] Dickson A, Yutuc E, Thornton CA, Dunford JE, Oppermann U, Wang Y, Griffiths WJ. 2023 HSD3B1 is an oxysterol 3β-hydroxysteroid dehydrogenase in human placenta. Figshare. (10.6084/m9.figshare.c.6502556) PMC10154939 37132223

